# Attitudes Toward Sexual Orientation and Gender Identity in Online Multiplayer Gaming Spaces

**DOI:** 10.1177/00332941231153798

**Published:** 2023-01-23

**Authors:** Laura E. Gillin, Margaret L. Signorella

**Affiliations:** Department of Psychology, 52501 The Pennsylvania State University, Brandywine, Media, USA

**Keywords:** gaming, video games, online multiplayer gaming, discrimination, gender, gender identity, sexual orientation

## Abstract

Online video game communities can provide a sense of belonging and support for marginalized people, while at the same time, can be rife with prejudice and discrimination. This study aimed to assess the prevalence of both positive and negative comments about sexual orientation and gender identity during online gaming, and to test the hypothesis that LGBTQ+ people witness or experience more prejudice than do heterosexual and cisgender persons. An online survey, distributed on social media sites and a psychology subject pool, included rating scales and open-ended questions on game-related conversations. Respondents (*N* = 185) provided negative examples made by others more frequently than positive ones and attributed serious comments to themselves versus jokes and offhand comments to others. Across all respondent gender identities, the targets of the negative comments by others were almost always LGBTQ+ persons. These results bolster critiques of online gaming environments as hostile to members of marginalized groups.

## Introduction

Video games continue to be both popular and controversial. In its recent industry summary, the Entertainment Software Association (2022) reported that 66% of the United States population plays video games and experiences many benefits, such as fun, stimulating and relaxing activity, and connections to others. Researchers are examining whether gaming has cognitive and mental health benefits (e.g., Boot et al., 2011; Colder Carras et al., 2018). At the same time, there are ongoing concerns about the potentially toxic or harmful aspects of gaming, from addiction to harassment to violence (e.g., Ferguson & Colwell, 2017). The incidences of biases in game content and toward players based on gender, race, and sexual orientation, have long been a focus of discussion and alarm (e.g., Shaw, 2015). In gaming contexts, however, there is less systematic published attention to the incidence and nature of homophobia (Shaw, 2015) and even less on transphobia (see Ballard & Welch, 2017, for an exception). In this research, therefore, we examined the online gaming environment experienced by players of differing gender identities and sexual orientations.

### Online Multiplayer Gaming

One especially prominent subcategory of video games is those played online with live persons worldwide. Communication between players during online gameplay can be facilitated by text-based chat boxes or microphones and headsets. Kowert et al. (2014) analyzed interviews with 2551 persons in Germany about gaming. In their sample, 35% indicated that they played online games. Many different topics can be explored in online games, ranging from what might be viewed as the stereotypical (or “core,” e.g., Cary & Chasteen, 2021) action and first-person shooter games to role-playing games to sports to puzzles (e.g., see Minor, 2022).

Online communities can offer a certain degree of anonymity, which can build a safe online environment, as Jenny Sundén (2009) described in her ethnographic study. Sundén, a member of an LGBTQ+ guild in the popular massively multiplayer online game (MMOG) *World of Warcraft* (Blizzard Entertainment, 2020), likened joining this LGBTQ+ space to coming home: “Sharing a passion for games, as well as sharing experiences of moving through life, and through the game as non-straight, forms a clear sense of togetherness and belonging” (p. 4).

Online gaming anonymity can also allow negative behaviors to flourish. The tendency for behavioral disinhibition to occur online has been well documented (e.g., Gray, 2014; Suler, 2004). Although disinhibition does not always lead to adverse outcomes, there are many well-known problem behaviors online, such as abuse, stalking, and trolling (e.g., Kowert, 2020; Mantilla, 2015; Paananen & Reichl, 2019; Suler & Phillips, 1998). Higgin (2015) further argued that technological advances in gaming interactions, coinciding with gaming population diversification, have led to “an explosion of offensive behavior and language which had previously been relatively isolated, contained, or hidden” (p. 4).

Salter (2017) said that “the encoding of… gender norms into online platforms has given these misogynist strands of geek culture a position of technological hegemony” (p. 248), and the impact of this hegemony extends to other marginalized groups and their intersections (e.g., Gray, 2018). Higgin (2015) characterized the “architecture of online gaming” as “vicious enforcement of White heterosexual and masculine normativity fueled by technologized anonymity” (p. 2). For example, Alexis Pulos (2013) wrote about heteronormativity in *World of Warcraft (WoW)*. Pulos criticized Blizzard (2020), the game developer, for regulating sexuality and sexual expression within their game. Pulos cites *WoW* forum posts in which users claim that sexual orientation has no place in the community. Other users dismissed the use of homophobic and transphobic slurs as “gamer lingo” (Pulos, 2013). These justifications of discrimination, Pulos claimed, painted the *WoW* community as intolerant and prejudiced, creating an uncomfortable or even unsafe environment for LGBTQ+ players.

### Previous Research on Biases in Gaming

Much of the previous research on biases in gaming has focused on the games' natures. Researchers and game enthusiasts have identified the narrow ranges of game types, themes, and identities. The focus is on white, male, cisgender, heterosexual players who are assumed to be the core audience (e.g., Dill et al., 2005; Paaßen et al., 2017; Richard, 2017; Stermer & Burkley, 2015).

Furthermore, there continues to be less research documenting the prevalence and experiences of individuals who play games but do not fit the gaming stereotype of a white cisgender heterosexual man (Gray, 2018). Most work to date has examined sexism. Racism and homophobia are gaining increased and needed attention (e.g., Cary & Chasteen, 2021).

Shaw (2015) has drawn attention to several gaps in the literature. She noted, for example:

The homophobia of online gaming spaces and the use of hate speech to police physical and virtual play has been discussed repeatedly, although often by collapsing it with online misogyny and rarely looking at its intersection with racism. (p. 65)

Shaw established that despite continued discussion of online homophobic harassment, there was a lack of research published in journals, with the focus tending to be on misogyny. Shaw searched gaming journals using gender identity and sexuality keywords and found few articles. Shaw concluded that “there is LGBTQ game research out there, but less than one would hope in 2015” (p. 71). For example, Shaw (2015) and later, Cary and Chasteen (2021), had to cite a survey about gay video game players that apparently has never been published. We could only find the results on the researcher’s now-defunct website through the Internet Archive (Rockwood, 2006) and in abbreviated fashion in the tech magazine *Engadget* (Sliwinski, 2007). The problem of harmful online gaming behaviors, either in general (e.g., Kowert, 2020) or specifically related to gender or sexuality (e.g., Henry & Powell, 2018), remains under-researched.

Systematic investigations of the experiences of trans individuals and possible transphobic behaviors in gaming are similarly sparse. Transphobia is not a new problem, either in the US (e.g., Nagoshi et al., 2008) or in other countries (e.g., Cabrera et al., 2022), but we know of only one study that included trans individuals in the gaming arena (Ballard & Welch, 2017; US sample). At the same time, there also appears to be a rise in prejudice against and discrimination toward transgender persons in the US. NBC News reported in March 2022 that there had been substantial increases in the first quarter of 2022 in legislation that targeted transgender individuals (Lavietes & Ramos, 2022). Specifically, they report that compared with 2019, when 37% of the anti-LGBTQ legislation was anti-transgender, over half the comparable legislation in 2021 and the start of 2022 were anti-transgender. Thus, the push to limit protections for transgender individuals in the US, although noticeably present in 2019, has increased. These data suggest that more attention is warranted to the description and impact of anti-transgender behaviors in gaming.

### Minority Stress and Microaggressions

If the gaming environment is noticeably hostile for LGBTQ+ individuals, there are clear mental health implications through either blatant or subtle discriminatory experiences. Meyer’s (e.g., 2003) concept of minority stress refers to “the excess stress to which individuals from stigmatized social categories are exposed as a result of their social, often a minority, position” (p. 675). Meyer’s theory has been extended to sexual minorities (e.g., Ramirez & Galupo, 2019). Two aspects of Meyer’s (e.g., 2003) theory are particularly relevant to LGBTQ+ players' gaming environment and experiences. Meyer proposed that stress could result from experiencing harmful events and anticipating those negative experiences, both of which are associated with mental health challenges.

The literature on microaggressions similarly shows that exposure to such behaviors has mental health consequences (e.g., Sue et al., 2007; Williams, 2020). Initial research on microaggressions focused on the experiences of persons of color, in which “…brief, everyday exchanges…send denigrating messages to people of color because they belong to a racial minority group” (Sue et al., 2007, p. 273). Nadal and colleagues (e.g., Nadal, 2019; Nadal et al., 2016) have identified microaggressions faced by LGBTQ+ individuals, such as showing discomfort in the presence of LGBTQ+ individuals, pronoun errors, and discounting of transphobia.

### Present Study

This research focused on documenting online game players' interactions relating to sexual orientation and to gender identity, thereby replicating and extending previous work to transgender and nonbinary individuals. Another focus was whether the experiences differed by the players’ sexual and gender identities. We build on the recent work by Ballard and Welch (2017), Cary et al. (2020), and Cary and Chasteen (2021), conducted with US and Canadian participants. We extended the work by Cary and colleagues to consider the game players' own gender identities and sexual orientation. We extended the work of all by recruiting a player sample that has a higher percentage of gender-diverse game-players than in previous work, by separating gender identity and sexual orientation as possible areas of negative as well as positive behaviors in online gaming, and by including concepts from the study of microaggressions.

Individuals who played online games were recruited for a study on LGBTQ+ experiences in online gaming. The online survey included rating scales and open-ended questions about the frequency by valence (positive or negative) and type (offhand comments, jokes, and serious discussions, based on the literature on microaggressions) of comments related to sexual orientation and gender identity. We asked about the frequency of perceived negative and positive comments. Both types have been documented in studies of sexism, misogyny, racism, and homophobia (e.g., Cary & Chasteen, 2021), but persons engaging in microaggressions can potentially perceive their comments as positive, whereas the target experiences negativity. We asked participants about their own and others’ behaviors, and about their own gender and sexual orientation.

### Hypotheses

This survey tested the hypothesis that LGBTQ+ persons would witness or experience more discrimination than heterosexual and cisgender persons (https://osf.io/vb72a). The preregistered hypothesis did not specify the operationalizations of the discriminatory behaviors, although these are implicit in the measures used. Based on the measures, the specific forms of discrimination potentially measured in this study would be the following:

1. Participants would report a higher frequency and more examples of negative than positive comments about sexual orientation or gender identity;
2. Participants would report more offhand comments and jokes than serious discussions about sexual orientation or gender identity;
3. LGBTQ+ respondents would report fewer serious discussions, more jokes and offhand comments, and more negative than positive comments and examples than heterosexual cisgender respondents.

No specific hypotheses were preregistered concerning comments or examples by self versus others. After the preregistration of the study, Cary et al. (2020) showed in a gaming context that respondents were more likely to report prejudiced statements by others than by themselves. Given the possibility, therefore, that individuals might engage in self-protective or socially desirable responding, a post hoc hypothesis was included.

4. Negativity may be more likely to be reported in others than self. Serious discussions or positivity may be more likely to be reported in self than others.

Therefore, this study aimed to assess the prevalence of both positive and negative comments about sexual orientation and gender identity during online gaming, and to test the hypothesis that LGBTQ+ people witness or experience more prejudice than do heterosexual and cisgender persons.

## Method

### Participants

Potential participants had to be 18 years of age or older and have played an online video game in the last 6 months, requirements specified on all recruitment materials and consent forms. We relied on self-report in both cases to confirm that these two requirements were met.

Recruitment occurred through two different methods: social media postings and a multi-campus psychology subject pool that included introductory social science students from several public university campuses in the mid-Atlantic region of the United States. The processes used for recruitment of these two samples are detailed in the procedure. Individuals recruited through social media were not compensated for their participation; those recruited through the subject pool received course credit. Information on the geographic locations of the social media participants was not collected.

The final convenience sample included 185 respondents for whom an identity group, based on gender identity and sexual orientation, could be assigned. The approach and steps we used to create the identity groups are described in the procedure.

The age distribution of the participants is based on 184 participants, as one person did not provide an age. One-half of the participants were between the ages of 18 and 24 (*n* = 93, 51%). Most of the rest (*n* = 72, 39%) were between 25 and 40. The remaining 10% included 16 participants who were between the ages of 41 and 50 and three who were older than 50 years of age.

Almost all the 185 participants answered the ethnicity question (98%). Among these 181 participants, the majority were White (*n* = 146; 80.7%). Of the remaining participants, five (2.8%) were Black or African American, nine (5.0%) were Asian, six (3.3%) were Hispanic/Latina/o/e/x, 14 (7.7%) were multiracial, and one (0.6%) was Native/Indigenous.

### Materials

Data were collected via a survey hosted on Qualtrics. There were two versions, one for participants recruited from the subject pool and one for those from social media sources. The only difference was the consent form, in which the subject pool respondents were informed about potential class credit. The survey contained qualitative and rating scale questions on online video game experiences within the last 6 months.

The survey opened with two general questions about game playing and behavior to ensure the respondents participated in relevant games and interacted with other players. The first question asked “Which online multiplayer games have you played most in the last 6 months? List up to three.” The next question was about the frequency of interaction during gaming. We focused on spoken and written communications in the rating of interaction frequency; specifically, “How often do you interact (e.g., text chat, headsets, etc.) with other players when playing online multiplayer games?”

Participants were then asked questions about their own online behavior and the behavior of others (actor) both for sexual orientation and transgender, gender-non-conforming, or other non-binary gender identities (content), for a total of 28 questions (8 open-ended and 20 rating). The questions were presented in four sets: target of other players and sexual orientation content first; followed by the target of self and sexual orientation content; target of other players and transgender, gender-non-conforming, or other non-binary gender identities content; and target of self and transgender, gender-non-conforming, or other non-binary gender identities content. Within each target X content set, the questions were the rated frequency of topic mentions by type (jokes, offhand comments, serious comments); the rated frequency of positive content mentions; examples of generally positive jokes, offhand comments, or serious discussion points on the content; the rated frequency of negative content mentions; examples of generally negative jokes, offhand comments, or serious discussion points on the content.

The questions on the survey were developed based on the experiences of frequent gamers, as discussed in case studies, ethnographic studies, and online forums or social media discussions about gaming. Individuals with some psychological methods background, diverse in gender, sexual orientation, ethnicity, and gaming experience, reviewed the entire survey and provided feedback before IRB submission. The sets of questions about the frequencies of comment types (jokes, offhand, and serious) were also based on the work of Nadal and others on microaggressions. For example, Nadal’s (2019) Sexual Orientation Microaggressions Scale contains a component (“Heterosexist Language,” pp. 1408–1409) that captures the experiences of insensitive jokes and comments. The four clusters of questions about comment types had acceptable to good internal consistency reliabilities (0.69, 0.81, 0.82, & 0.82). The four sets of valence questions had only two items, so intercorrelations were examined and were all significant and in expected directions (see also Cary et al., 2020; Cary & Chasteen, 2021). The patterns of intercorrelations in general showed convergent and divergent validity.

The last four questions of the survey assessed demographics, asking the participants to provide race/ethnicity, age, gender, and sexual orientation. All but age were open-ended. We chose to use open-ended questions to allow participants to self-identify without presenting answers that might influence their responses (e.g., see Lowik et al., 2022). Research on identities has recognized that individuals do want the opportunity to express their identities and can be frustrated with commonly used labels (e.g., Galupo et al., 2016). The complete redacted questionnaires are available (https://osf.io/jt47w/).

One minor unintentional variation in wording on both questionnaire versions was present. The response options for all the questions about frequency were presented from most frequent to least frequent, and four of the five options were identical on all rating scale items (fairly often, about half the time, not very often, and never or almost never). The first option on the rating scale was sometimes labeled “every time or almost every time” and at other times was labeled “always or almost always.” These two phrases appear to be synonymous.

All rating questions were coded for the analyses so that higher numbers indicated more frequent actions, from zero (0) indicating never or almost never to four (4) indicating the highest frequency. The data are available here: https://osf.io/wq4zc/.

### Procedure

The study was preregistered prior to the submission of the study to the IRB (https://osf.io/vb72a).

#### Recruitment

The solicitation of participants occurred via two different methods following university IRB review and designation as exempt. Students at the authors’ university were recruited through the cross-campus psychology subject pool using the standard procedure, posting a short description with requirements on the pool system. To recruit from a more general population, a description of the study and a link to the survey was posted on several social media sites that are used to solicit survey responses (Facebook, Reddit/r/SampleSize [a subreddit specifically for posting surveys], Tumblr, and Twitter). After consent was obtained, participants were asked to complete the survey. Upon completion, all participants were shown a “thank you” message and provided with a link to a 24-hour LGBTQ+ hotline.

Data were collected over 7 weeks. The survey went live on the subject pool and some social media (Facebook, Tumblr, and Twitter) on 2019 January 23 and on Reddit (r/SampleSize) on 2019 January 30. The link to the survey was reposted on Reddit and Tumblr on 2019 February 13. Both versions of the survey were closed on 2019 March 13.

#### Data Screening

A total of 493 individuals entered the online surveys. The multi-campus subject pool initially recruited 30 individuals, but two who agreed to participate did not answer any questions. Social media links brought in 463 individuals; of those, one declined to participate on the consent page, two did not answer the request for consent, one answered the first set of questions before exiting the survey, and 231 agreed to participate but did not answer any of the questions. In summary, 51.9% (256/493) answered some or all questions.

Out of those remaining 256 participants who completed some or all of the survey (28 from the subject pool and 228 from social media), there were further data lost from 71 participants. The first issue was that three participants failed validity checks on one of the open-ended questions. The most extensive data loss occurred with the participants whose surveys were incomplete (*n* = 68). These respondents stopped at various points before the end of the survey or gave uncodeable answers to the gender identity and sexual orientation questions, thus precluding categorization into an identity group. To examine whether those who completed the survey (*n* = 185) were different from those whom we excluded (*n* = 71), we compared responses to the early question on the frequency of interaction with others during online gaming. The two groups were not significantly different in their reported frequency of interacting with others during online games (*p* = .78).

#### Identity Groups

It was necessary to group participants so that there would be large enough groups to make comparisons. In the explanation that follows, we tried to be aware of the issues outlined by Lowik et al. (2022) that are present when grouping persons based on open-ended responses about gender, gender identity, and sexuality. A major goal in grouping was to represent identities that were likely or not to be targets of prejudice in the gaming context. We reviewed the open-ended responses to the gender and sexual orientation questions by the 185 participants who answered both and then as a first step made groupings based on the two questions separately.

The responses to the gender question led to three initial groupings: 97 (52.4%) men, 63 (34.1%) women, and 25 (13.5%) nonbinary persons. Individuals were first grouped with men if they gave their gender as male or man, cisgender or cis male or man, or trans or transgender male or man. Individuals were first grouped with women if they gave their gender as female or woman, cisgender or cis female or woman, or trans female or woman. Individuals were classified with nonbinary persons if they responded with something other than a traditional binary gender (e.g., nonbinary [including nonbinary male or nonbinary female], agender, genderfluid, genderqueer, non-conforming).

The responses to the sexual orientation question resulted in just under half of the participants grouped as heterosexual, with 79 (43%) participants reporting their sexual orientation as heterosexual or straight. The other 106 (57%) participants were initially categorized as not heterosexual, but note that this othering was not an acceptable final grouping. The most frequent identities stated by these latter participants were gay, bisexual, lesbian, pansexual, asexual, and queer.

We needed to be able to represent both gender and sexual orientation in the participant groups, but there were not enough persons in specific marginalized identities to have each in separate groups. Therefore, we grouped the participants as follows into five identity groups. The GBTQ+ men included 43 cisgender and transgender men who identified as any non-heterosexual identity. The LGBTQ+ women included 38 cisgender and transgender women who met the corollary criteria. There were very few respondents who self-identified as transgender men or women, thus precluding a separate group. But all who self-identified as transgender men or women also identified with a frequently marginalized sexual orientation. Therefore, the transgender men and women were included in the two binary groupings in which there is a higher probability of being a target of prejudice based on gender and sexuality. The nonbinary group was comprised of 25 individuals who identified as any gender identity beyond the binary and with any frequently marginalized sexual orientation. The heterosexual men included 54 men who identified as heterosexual or straight. The heterosexual women group included 25 women who met the corollary criteria.

### Open-Ended Response Coding

The responses to the open-ended questions asking for examples of positive and negative comments were reviewed by individuals representing a diversity of gender identities and sexual orientations to allow the contextual and cultural frameworks of the comments to be considered. These initial reviewers included the authors and two research assistants of one of the authors. Neither of the RAs was otherwise involved in the present study.

The process used for this first cycle was iterative thematic coding (e.g., Guest et al., 2012, Ch. 3). Each reviewer was given a printout or data file of the relevant open-ended responses. No other information about the participants was included. Reviewers made notes on the themes or types of responses that were given for a particular question. The themes that emerged for the positive examples were accepting the sexual orientation or gender identity of others, standing up to players spreading negative comments, LGBTQ+ players connecting, and educating/seeking education on LGBTQ+ topics. The themes for negative examples were insults, threats, slurs, general wishes of harm, and invalidation of identity. These latter types of themes are consistent with research on microaggressions and toxic gaming (e.g., Kowert, 2020; Nadal, 2019). Using these themes, pairs of coders from among the reviewers then coded questions; however, the reliabilities for the pairs of coders did not reach adequate levels.

Reviewers’ discussion on the lack of agreement revealed that although specific themes were not reliably identified, themes tended to be consistently positive or negative. We therefore decided to only code positive or negative overall content, the focus of the hypotheses. The reviewer discussions also identified two other areas that needed to be coded. One was the target of negative comments about sexual orientation. Most negative comments were aimed at LGBTQ+ identities, but not all, so we included a code for the identity being targeted. Second were the various comments that did not fit the question being asked (off-topic comments or comments that were negative in response to a request for a positive example) and explicit statements that the respondent had no examples to offer.

For the final coding, responses to the four questions requesting examples of positive jokes, offhand comments, or serious discussion points were then coded into the following categories: positive toward LGBTQ+ identities or sexual orientation/gender identity in general, not positive, irrelevant, or none. Responses to the four questions requesting examples of negative jokes, offhand comments, or serious discussion points were coded into one of three categories: negative toward LGBTQ+ identities, negative towards cisgender/heterosexual identities, none. Responses were coded as none if the person answered explicitly, such as none, N/A, dashes, or statements such as “I can’t think of any” or “I never talk about…”

The authors coded all comments (Krippendorff’s alpha = 0.79). Disagreements were resolved by discussion. Those who left an item blank or gave an irrelevant response were not included in the open-ended question analyses reported in the results. Those answers (or nonanswers) were viewed as uninterpretable.

The comments for each open-ended question by identity group are in the supplemental materials (https://osf.io/bf846/). The comments were edited to remove any identifying information about respondents and redact slurs.

## Results

### Overview

An essential comparison for interpreting any differences in gaming behavior or experience by identity group was the frequency of interaction with other players to ensure that any differences in comment types and frequency are not due to baseline differences in commenting frequency. All but one of the 185 participants answered this question. A one-way between-groups ANOVA on the rating of how frequently the respondents interacted with other players showed no statistically significant differences among the identity groups, *F* (4,179) = 0.61, *p =* .66. The identity group means ranged from 2.2 to 2.6, which on the scale is between *about half the time* (2) and *fairly often* (3).

For the main analyses, ratings and open-ended coded responses were analyzed. As many comparisons were being made for each of the three main analyses (ratings of comment valence frequency, open-ended example codes, ratings of comment type frequency), Bonferroni corrections were applied to each group of tests. Critical values for the omnibus tests were .0036, .0006, and .0036, respectively. Additional Bonferroni adjustments were used for simple effects tests, pairwise comparisons, and goodness-of-fit tests when needed for follow-ups.

### Comment Valence and Examples During Gaming

The participants were asked to rate the frequency, during gameplay, of positive comments and negative comments that related to sexual orientation and to gender identity (transgender, gender-non-conforming, or other non-binary gender identities), by others and by themselves. After each rating (e.g., positive comments made by self about sexual orientation), participants were asked to give an example. These questions test the hypotheses that participants would report that negative comments were more frequent than positive comments about sexual orientation or gender identity; would report more examples of negative than positive comments about sexual orientation or gender identity; and that both patterns would be more likely to occur with LGBTQ+ participants than with heterosexual cisgender participants.

For the outcome variable of frequency ratings (from 0 = lowest frequency to 4 = highest frequency), an Identity Group (5: GBTQ+ men, LGBTQ+ women, nonbinary persons, heterosexual men, heterosexual women) × Content (2: sexual orientation, gender identity) × Actor (2: self, other) × Valence (2: positive, negative) mixed models ANOVA was conducted, with repeated measures on Content, Actor, and Valence (see Table 1).

**Table 1.** Means and Standard Deviations for Comment Frequency by Identity Group, Content (Sexual Orientation, Gender Identity), Actor (Self, Other), and Valence (Positive, Negative).

Comment Frequency	Identity Group				
	GBTQ + Men	LGBTQ + Women	Nonbinary	Heterosexual Men	Heterosexual Women
Sexual orientation by self					
Positive	1.43 (0.77)	1.00 (0.40)	1.25 (0.68)	1.23 (1.07)	1.28 (0.94)
Negative	0.45 (1.02)	0.21 (0.41)	0.04 (0.20)	0.51 (1.07)	0.60 (1.22)
Sexual orientation by other					
Positive	1.35 (1.19)	1.26 (1.16)	1.16 (1.14)	1.25 (1.24)	1.20 (0.97)
Negative	2.53 (1.26)	2.55 (1.33)	2.64 (1.44)	2.26 (1.47)	2.44 (1.23)
Gender identity by self					
Positive	2.80 (1.70)	3.55 (1.13)	3.13 (1.57)	2.23 (1.77)	3.40 (1.04)
Negative	0.27 (0.78)	0.21 (0.66)	0.22 (0.74)	0.58 (1.20)	0.36 (0.91)
Gender identity by other					
Positive	0.93 (1.20)	1.03 (1.33)	0.71 (1.16)	0.90 (1.27)	1.40 (1.19)
Negative	2.12 (1.56)	2.55 (1.55)	2.48 (1.62)	1.85 (1.60)	2.28 (1.43)

Note: The *n*s for each identity group ranged as follows: GBTQ+ men = 40–43 (out of 43); LGBTQ+ women = all 38 (out of 38); Nonbinary = 23–25 (out of 25); Heterosexual men = 48–53 (out of 54); Heterosexual women = all 25 (out of 25). Ratings could range from 0 to 4, with higher numbers indicating more frequent comments.

The four-way interaction was the highest order significant effect, *F* (8, 1263) = 3.39, *p* = .001. To follow up this interaction, the results for sexual orientation comments and gender identity comments were examined separately, as there were no predictions about any differences between these two topics.

The two content analyses (sexual orientation, gender identity) showed that the self-protective pattern predicted post hoc was present for both topics in valence X actor interactions. Respondents indicated that other persons made negative comments more frequently than positive ones, whereas they made positive comments more frequently than negative ones. For sexual orientation content, this valence × actor interaction, F (1, 534) = 143.5, *p* < .0001, was not modified further. For gender identity content, the valence × actor interaction showing the self-protective response pattern, *F* (1, 519) = 371.6, *p* < .0001, was subsumed by a three-way interaction that added identity group, *F* (4, 519) = 4.92, *p* = .0007. The identity group factor did not alter the self-protective pattern, but rather was significant because heterosexual men reported making less frequent positive gender identity comments than did the two groups of women (LGBTQ+, heterosexual).

Table 2 displays the frequencies of the coded comment types for each open-ended question. Respondents were asked for positive and negative examples of both their own and others’ comments regarding sexual orientation and gender identity. As there was only one significant difference in types of responses by identity group, which will be discussed below, Table 2 does not include identity group breakdowns. In addition, when asked for positive examples, a few respondents gave examples that were coded as not positive. These small numbers of instances were combined with the respondents who indicated they did not have any positive examples. Chi-Square goodness-of-fit tests were used to test whether the frequencies of types of responses were significantly different from a 50/50 split.

**Table 2.** Comment Type Frequency by Content (Sexual Orientation, Gender Identity), Actor (Self, Other), and Valence (Positive, Negative).

Example Valence	Comments About Sexual Orientation		Comments About Gender Identity	
	By Self	By Others^a^	By Self	By Others
Positive prompt examples coded as				
Positive	60%	47%	50%	27%
Not positive or none	40%	53%	50%	73%
*N*	130	129	107	100
Negative prompt examples coded as				
Negative toward LGBTQ+	21%	89%	11%	67%
Negative toward cisgender heterosexual	9%	0%	1%	0%
None	70%	11%	88%	33%
*N*	101	148	84	113

^a^There was a significant association between comment code type and identity group, χ^2^ (4, *N* = 129) = 21.1, *p* < .001. LGBTQ+ women and GBTQ+ men reported hearing more positive comments about sexual orientation (18/29 and 20/28, respectively), and heterosexual men reported hearing fewer (7/36), compared with the other identity group persons.

The self-protective pattern observed in the ratings was apparent for all four prompts asking for examples of negative comments. Significant majorities (70% for sexual orientation; 88% for gender identity) indicated they personally made no negative comments. The opposite pattern occurred when asked about comments by others. Significant majorities indicated that they saw others making negative comments (89% for sexual orientation; 67% for gender identity).

For the four prompts soliciting positive examples, the self-enhancing pattern appeared most prominently in one of the four. When asked for examples of positive comments about gender identity by others, there was a significant deviation from a 50/50 result, with 73% unable to cite a positive example that another player made. For examples of their own positive comments, about half provided an example and half did not. Identity group differences emerged on the prompt for examples of positive comments by others about sexual orientation. Overall there was no significant deviation from a 50/50 (positive vs. none) pattern, but LGBTQ+ women and GBTQ+ men reported hearing more positive comments, and heterosexual men reported hearing fewer, compared with the other identity groups (see Table 2 note).

### Comment Types During Gaming

The participants were asked to rate the frequency during gameplay of jokes, offhand comments, and serious discussions, by others and by themselves, that were related to sexual orientation and gender identity (transgender, gender-non-conforming, or other non-binary gender identities). This comparison tests the hypotheses that participants would report more offhand comments and jokes than serious discussions about either sexual orientation or gender identity, and that the LGBTQ+ respondents would be more likely to be exposed to this pattern than would the heterosexual cisgender respondents.

An Identity Group (5: GBTQ+ men, LGBTQ+ women, nonbinary persons, heterosexual men, heterosexual women) X Content (2: sexual orientation, gender identity) × Actor (2: self, other) × Comment Type (3: jokes, offhand, serious) mixed models ANOVA was done on the frequency ratings (from 0 = lowest frequency to 4 = highest frequency), with repeated measures on Content, Actor, and Comment Type (see Table 3). There were four significant two-way interactions.

**Table 3.** Means and Standard Deviations for Comment Frequency by Identity Group, Content (Sexual Orientation, Gender Identity), Actor (Self, Other), and Comment Type (Joke, Offhand, Serious).

Comment Frequency	Identity Group				
	GBTQ + Men	LGBTQ + Women	Nonbinary	Heterosexual Men	Heterosexual Women
Sexual orientation by self					
Joke	1.30 (1.47)	1.22 (1.18)	1.20 (1.26)	0.61 (0.92)	0.32 (0.56)
Offhand	0.93 (1.22)	1.22 (1.17)	1.04 (1.17)	0.45 (0.87)	0.38 (0.58)
Serious	1.05 (1.21)	1.37 (1.24)	1.28 (1.34)	0.52 (0.75)	0.50 (0.78)
Sexual orientation by other					
Joke	2.42 (1.24)	2.24 (1.30)	2.20 (1.22)	1.70 (1.24)	2.04 (1.24)
Offhand	2.26 (1.00)	1.68 (1.07)	2.08 (1.04)	1.46 (1.27)	1.48 (1.24)
Serious	0.70 (0.77)	0.65 (0.86)	0.75 (0.94)	0.67 (1.06)	0.57 (0.84)
Gender identity by self					
Joke	0.33 (0.79)	0.54 (1.02)	0.72 (1.14)	0.26 (0.56)	0.08 (0.28)
Offhand	0.25 (0.54)	0.72 (1.09)	0.96 (1.10)	0.26 (0.59)	0.08 (0.28)
Serious	0.65 (1.14)	0.91 (1.16)	1.25 (1.36)	0.46 (0.64)	0.04 (0.20)
Gender identity by other					
Joke	1.40 (1.24)	1.53 (1.37)	1.44 (1.29)	0.70 (0.92)	1.08 (1.15)
Offhand	1.19 (1.31)	0.92 (0.88)	1.20 (1.22)	0.57 (0.84)	1.04 (1.16)
Serious	0.44 (0.59)	0.41 (0.80)	0.52 (0.82)	0.32 (0.73)	0.50 (0.88)

Note: The *n*s for each identity group ranged as follows: GBTQ+ men = 40–43 (out of 43); LGBTQ+ women = 34–38 (out of 38); Nonbinary = 24–25 (out of 25); Heterosexual men = 52–54 (out of 54); Heterosexual women = 23–25 (out of 25). Ratings could range from 0 to 4, with higher numbers indicating more frequent comments.

The hypothesized comment type pattern appeared in a form similar to that shown in the valence ratings and open-ended responses. Specifically, there was a self-enhancing pattern of respondents claiming that other persons made jokes and offhand comments more frequently than serious comments, while claiming that their own comments were more frequently serious rather than joking or offhand, *F* (2, 1938) = 110.4, *p* < .0001.

The only significant identity group effect was an interaction with actor, *F* (4, 1939) = 8.70, *p* < .0001. Although all respondents reported that others made more frequent comments overall than they did themselves, the LGBTQ+ women and nonbinary persons reported making more comments overall than did GBTQ+ men, heterosexual men, and heterosexual women.

The other two interactions involved actor, content, and comment types. Actor and content interacted, F (1, 1939) = 12.6, *p* = .0004, such that respondents rated other persons as commenting more frequently when the topic was sexual orientation than when it was gender identity. Content and comment type interacted, F (2, 1939) = 14.3, *p* < .0001, such that there were more frequent jokes and offhand comments concerning sexual orientation versus serious comments concerning gender identity.

## Discussion

The primary goals of this study were to assess the frequency of comment types encountered by individuals while playing online games and to extend previous sexual orientation-focused work to transgender, gender-non-conforming, or other nonbinary gender identities. We hypothesized that LGBTQ+ individuals would encounter more discriminatory environments in gaming. Specifically, it was hypothesized that the discrimination would be in the form of less frequent serious comments and more frequent negative ones concerning sexual orientation and gender identity. We also predicted that LGBTQ+ persons would be more likely to experience these patterns.

The results provided partial support for the hypotheses in the frequency and nature of comments reported to have been made by others. Reports of negative comments by others about LGBTQ+ sexual orientations and identities dominated the valence ratings and the coded open-ended responses. Everyone described hearing more negative than positive comments from others about sexual orientation and transgender, gender-non-conforming, or other nonbinary gender identities. One possible ambiguity in the ratings of negativity about sexual orientation could be whether the reported negativity was aimed equally at any orientation. After each frequency rating, however, examples were requested. Almost all the negative examples were coded as being directed at LGBTQ+ individuals. Thus, the prediction that there would be more negative than positive comments concerning LGBTQ+ persons was supported by the reports of what others were saying. This pattern is similar to recent data from surveys by Cary and colleagues (Cary et al., 2020; Cary & Chasteen, 2021). Survey participants from the US and Canada reported others making prejudiced comments about gender, race, and sexual orientation (Cary et al., 2020). Prejudiced comments about these social categories were more frequent in role-play or action game interactions than in puzzle games or face-to-face gameplay in a US sample (Cary & Chasteen, 2021).

What emerged also was support for the post hoc prediction, seen in the Cary et al. (2020) results, that self-protecting or self-enhancing behaviors might result in respondents emphasizing their own positive behaviors, while attributing negative ones to others. A consistent pattern observed across questions was that respondents viewed their own behavior as more positive, less negative, and more serious than that of others. The present data cannot elucidate whether this is an accurate perception or a reluctance to admit to less than admirable actions. Nonetheless, for both sexual orientation and gender identity, the reported frequencies of negative comments by others were higher than positive comments by other persons, similar to the pattern reported in Cary et al. (2020, p. 629).

One prediction that was not supported, however, was the expectation that LGBTQ+ persons might report hearing prejudicial comments more frequently than heterosexual men and women would. The few comparisons in which identity group had a significant impact did not form any consistent pattern. In the Ballard and Welch (2017) study, the LGBT participants reported greater problems than did heterosexual participants in some but not all categories of cyberbullying included in their survey (specifically; lies, sexual harassment, sexual pursuit, group exclusion, Table 4, p. 479). Given the paucity of research on this issue, more data are clearly needed.

Even if LGBTQ+ individuals are experiencing the same frequencies of comments as are cisgender heterosexual individuals, all are unfortunately being exposed to gaming environments with much negativity almost exclusively directed at LGBTQ+ persons. In the Ballard and Welch (2017) study, the heterosexual participants were more likely than the LGBT participants to report bullying others. Harmful impacts of minority stress (e.g., Meyer, 2003) and microaggressions (e.g., Nadal et al., 2016) have been identified. Cisgender heterosexual individuals hearing the slurs, insults, and “jokes” aimed at those with different identities could also be impacted, including learning that such behavior is normative (e.g., Cary et al., 2020) or worse, acquiring models for engaging in harassment of LGBTQ+ players.

### Broader Implications for Gaming

The critique that the gaming environment favors young white male cisgender heterosexuals is an important one but is an even broader equity issue (e.g., Paaßen et al., 2017). Although controversial, games have the promise to provide not only entertainment but also positive impacts on cognition and mental health (e.g., Boot et al., 2011; Colder Carras et al., 2018). As these matters are being debated, recent data show the extent to which video games are a common human activity. In their 2022 report, the Entertainment Software Association reported that 66% of persons in the US play video games “at least weekly” (p. 3). There are now almost as many women and girls playing as there are men and boys. Pew Research Center data (Brown, 2017; Perrin, 2018) similarly show that gaming is popular, and although still showing gender disparities, is not limited to young white men—or, as Brown’s (2017) report title conveyed: “Younger men play video games, but so do a diverse group of other Americans.”

### Limitations and Future Directions

As the present data are self-report, we do not know if the respondents were downplaying or overstating the severity of the problem. Even content analyses of posts, such as in the recent study of the Reddit gaming community (Maloney et al., 2019), could be vulnerable to self-presentation or social desirability biases. The patterns do, however, replicate recent research and are consistent with case studies and news reports. To cross-validate these findings experimentally would be an important next step. An example would be the Kuznekoff and Rose (2013) experimental manipulation of a gaming partner’s gender during live online gaming interactions. Their results showed that women received more negative responses than did men. Similar studies in popular online gaming arenas could provide information that may be less impacted by social desirability or other biased self-report responses.

Due to the lack of variability across participant race and ethnicity, this study could not consider how LGBTQ+ identities intersect with racial and ethnic identities during online gameplay. The sample of participants was also not varied across age, with only 10.3% of participants over 40. Cluster sampling of diverse gaming communities is needed to represent the experiences of all gamers.

Missing data are also a potential complication in interpreting the frequency of different comment types. One type of missing data is the failure to complete the survey. As far as we can tell from comparing the frequency of gaming interaction between those who completed the survey and those who did not, there was no significant difference. There could be another important difference that we have not assessed between those who completed or did not complete the survey. The other type of missing data occurred because some respondents did not answer one or more open-ended questions, even though they completed the rest of the survey. These nonresponses then become an issue in interpreting the nature and frequency of the different types of examples provided to the open-ended questions. Most crucially, are participants who leave a question blank intending this to be the same as those who wrote in “none” or “N/A” or “can’t think of any” when asked for examples of positive or negative statements? Or are they simply respondents who prefer not to type in answers to questions that they just answered in general on a rating scale? We examined the patterns of open-ended questions left blank, and those patterns suggest fatigue at typing in answers as a most likely explanation, as there were generally increasing numbers of blanks toward the end of the questionnaire.

## Conclusions

Research reviewed here supports the conclusions that online games are popular, have the potential to have positive impacts beyond entertainment, and engage individuals from diverse identities. Yet, as shown in the present and earlier research, members of marginalized groups are more likely to be exposed to negative environments targeting their identities. The present study replicated and extended findings from previous work on gender, misogyny, race, and sexual orientation and showed that both heterosexual and LGBTQ+ players are exposed to negative discourse related to both sexual orientation and gender identities, potentially resulting in at worst a toxic environment and traumatic experiences and at best providing a deplorable example.

There are unresolved debates about how to manage behaviors in many social media arenas, and as yet there is no consensus on how to make online environments open as well as safe (e.g., Kowert, 2020; Ortutay & Wiseman, 2020; Vitak et al., 2017). Kowert (2020) has suggested that gamers confronting bad behaviors could be an effective strategy. For example, on April 1, 2019, the Reddit forum on games (r/Games) closed to plea for stopping the “misogyny, transphobia, homophobia, racism or a host of other discriminatory practices” regularly displayed in the forum (r/Games mod team, 2019). This call to action was consistent with the Maloney et al. (2019) analysis of the subreddit r/gaming. Maloney et al. (2019) concluded their study with caution and optimism:

We do not underestimate the degree of change that is still required, or the level of entrenched misogyny that underpins female gamers’ experiences of marginalisation and harassment… [but] a pattern of other, healthier and more equal voices is playing out across this culture. (p. 99)

Norms in gaming that de-emphasize and police the harmful behavior are needed. Games and game-related sites have made some efforts in this direction (e.g., see the rules for the two *Reddit* gaming sites, which are delivered sternly but are not identical). A constant complaint, however, across many types of games, game sites, and social media more generally, is that if there are rules, they are not enforced effectively (e.g., Jones, 2016). For changes to encompass all marginalized voices, and rapidly enough, seems unlikely absent sites and games having common rules and enforcing them.
